# A causal deep learning approach to identifying metabolic signatures of cognitive and functional decline in alzheimer’s disease

**DOI:** 10.1038/s41598-025-32793-2

**Published:** 2026-01-03

**Authors:** B. Priyadarshini, John Sahaya Rani Alex

**Affiliations:** School of Electronics Engineering, Vellore Institute of Technology, Chennai, India

**Keywords:** Alzheimer’s disease, FDG-PET, Brain glucose metabolism, Cognitive decline, Functional impairment, Causal inference, MMSE, FAQ, Feature-wise attention mechanism., Diseases, Neurology, Neuroscience

## Abstract

Cognitive and functional decline in Alzheimer’s disease (AD) arises from disruptions in specific brain networks. Identifying the most affected regions is essential for understanding disease progression and developing targeted interventions. Fluorodeoxyglucose positron emission tomography (FDG-PET) offers a sensitive method for detecting early metabolic dysfunction, often before structural changes become apparent. We examined regional brain glucose metabolism in relation to cognitive performance and functional independence across cognitively normal individuals, those with mild cognitive impairment (MCI), and AD patients. Cognitive function was measured using the Mini-Mental State Examination (MMSE), and daily functioning was assessed via the Functional Activities Questionnaire (FAQ). Imaging and clinical data were obtained from the Alzheimer’s Disease Neuroimaging Initiative (ADNI). Structural causal modeling was used to identify brain regions with a strong causal influence on MMSE and FAQ scores. These causally validated regions were then used as input to the proposed FDG-PET-based Cognition Prediction Network (FDG CogNet), a deep learning model, which includes a feature-wise attention mechanism to dynamically weight each region’s contribution to prediction. Temporal, parietal, and hippocampal regions were most influential for cognitive performance, particularly in early stages of disease. Functional abilities were more strongly associated with executive and integrative regions, including the angular gyrus, temporal poles, posterior cingulate, and frontal cortices. Cerebellar regions showed compensatory activity in MCI but diminished in AD, suggesting reduced neural resilience. FDG CogNet achieved high predictive accuracy, with R² = 0.90 for MMSE and R² = 0.94 for FAQ, demonstrating that limiting inputs to causally relevant regions improved both performance and interpretability. These findings clarify stage-specific neural mechanisms in AD and show that combining causal inference with an attention-based deep learning model provides a powerful framework for accurate and interpretable prediction. This approach highlights the clinical utility of FDG PET for early diagnosis and suggests that timely, region-specific interventions offer the best opportunity to preserve cognitive and functional abilities in AD.

## Introduction

Alzheimer’s disease (AD) is a debilitating neurodegenerative disorder characterized by progressive decline in cognitive functions, including memory, attention, language, and executive abilities. This deterioration impairs daily functioning and independence^1^. Clinicians often rely on evaluation methods like the Mini-Mental State Examination (MMSE) and the Functional Activities Questionnaire (FAQ) to quantify these cognitive and functional deficits, providing valuable insights into disease severity^2^. However, these assessments provide a limited understanding of the underlying neural disruptions and mechanisms responsible for the observed cognitive deterioration^3^. As changes in brain structure and function often begin before noticeable cognitive symptoms emerge^4^there is a growing need to incorporate complementary biological approaches that can capture these underlying alterations. To address this need, neuroimaging modalities play a key role in Alzheimer’s research by offering biological perspectives that complement clinical evaluations. For example, structural Magnetic Resonance Imaging (MRI) helps identify brain atrophy, while functional MRI (fMRI) reveals activity patterns during cognitive tasks. On the other hand, Diffusion Tensor Imaging (DTI), a structural connectivity technique, provides insights into white matter integrity and neural communication pathways, while Positron Emission Tomography (PET) captures functional changes in brain metabolism^5^.

Among these valuable neuroimaging tools, PET is particularly noteworthy for its unique capacity to capture the brain’s molecular and metabolic processes. These ‘molecular processes’ encompass early biochemical changes, such as altered glucose metabolism, the abnormal accumulation of proteins like beta-amyloid or tau, and neuroinflammation mechanisms that precede visible structural damage and offer cellular-level insights into disease progression^6^. Specifically, PET imaging with 18 F-fluorodeoxyglucose (FDG) allows for detailed assessment of regional brain glucose metabolism, serving as a vital indicator of neuronal viability^7^. The strength of PET imaging lies in its ability to quantify and map metabolic activity across brain regions. When combined with clinical assessments, this enables the identification of region-specific abnormalities that are closely linked to distinct cognitive and functional impairments. This integrated approach is not only essential for enhancing the accuracy of diagnosis but also for unravelling the fundamental pathophysiological mechanisms that drive cognitive deterioration. Understanding the link between metabolic imaging and clinical symptoms is key to detecting early, region-specific changes in the brain. This connection enables timely interventions, more accurate monitoring of disease progression, and the development of targeted therapies for AD.

In this context, various studies have been investigated how structural and functional changes in particular brain areas correlate with cognitive performance measures. In healthy older adults, preserved brain structure is consistently associated with better global cognition. For instance, Endres et al.^8^ reported that larger grey matter and hippocampal volumes were associated with higher MMSE performance. Similarly, Murai et al.^9^ showed that reduced grey matter volume and abnormally short neural timescales in the Angular Gyrus were linked to poorer scores on the MMSE in AD. Longitudinal MRI evidence further indicates that superior episodic memory is supported by higher hippocampal activity and reduced hippocampal atrophy, reinforcing the hippocampus as a stable determinant of memory function across adulthood^10^Also, a Magnetoencephalography (MEG)-based study showed that the left supramarginal gyrus is a key node in cognitive processing, with elevated beta-band oscillatory activity strongly correlating with the severity of cognitive impairment. Beyond temporoparietal regions, the posterior cingulate cortex (PCC) and parahippocampal cortex are also implicated in memory, spatial cognition, language, and semantic integration. Altered structural and connectivity patterns in these areas have been frequently associated with cognitive decline in aging and AD^11,12^. Age-related alterations in hippocampal and temporal pole volumes further contribute to declines in episodic autobiographical memory in older adults^13^. These findings consistently highlight the need for region-wise analyses to elucidate how specific neural changes contribute to cognitive decline in AD. To enable a more systematic and powerful exploration of these intricate relationships, implementing standardized PET image pre-processing and region-level analysis allows the extraction of meaningful metabolic features that reflect underlying neuronal function^14^. Moreover, integrating neuroimaging features with clinical and demographic variables creates a structured tabular format that is robust for applying advanced computational models to decode region-specific contributions to cognitive decline.

Traditional statistical methods like Pearson and Spearman correlations, correlation matrices, and region-wise linear modelling have served as essential tools for identifying basic relationships between brain imaging features and cognitive scores^15^. However, the increasing complexity of multimodal AD datasets demands methods that can capture non-linear, multivariate interactions^16^. These challenges have led to the development of advanced computational methods designed to capture complex, non-linear relationships in the data. As a result, machine learning (ML) techniques have gained momentum for their ability to analyse and interpret rich, high-dimensional neuroimaging datasets.ML models, including Support Vector Machines (SVM) and Decision Trees, have been widely used as classifiers in neuroimaging datasets, primarily for distinguishing between AD stages or Clinical groups^17,18^. However, they have not been extensively applied in direct conjunction with cognitive scores to explore region-wise metabolic associations. Building upon these ML capabilities, Deep Learning (DL) models have emerged as a promising approach for capturing complex, interdependent feature relationships without requiring extensive manual intervention. Architectures like TabNet, Neural Oblivious Decision Ensembles (NODE), and Self-Attention and Intersample Attention (SAINT) overcome these constraints by capturing non-linear, interdependent feature relationships. Among these, Tab Transformers are particularly effective, as their self-attention mechanisms dynamically assess contextual relationships between features within each data instance, enabling the identification of subtle patterns in complex datasets^19^.

Despite progress in neuroimaging and ML, significant methodological challenges remain in identifying which regional metabolic abnormalities directly contribute to cognitive and functional decline in AD. Most existing studies rely on correlation-based methods to link regional brain changes with clinical scores. While these approaches can highlight associations, they fall short of establishing causal direction, limiting their ability to determine the brain regions that actively drive cognitive deterioration. Although causal inference methods have recently gained traction in neuroscience, their application has largely been confined to functional MRI studies focusing on network-level connectivity. In contrast, the use of causal modeling for PET image-derived features, particularly Standardized Uptake Values (SUVs), is still limited and underexplored. Additionally, while DL models such as Tab Transformers are increasingly used in biomedical tabular data, they generally treat all input features uniformly, lacking the ability to emphasize clinically relevant brain regions.

To overcome these limitations, this study introduces an end-to-end integrated framework that combines structural causal inference with a novel deep learning architecture. This approach is designed to enhance the prediction of cognitive and functional decline in AD by utilizing region-specific brain metabolism.

The significant contributions of this framework are,

- A Structural Causal Model (SCM) was applied to capture the causal relationships between regional brain metabolism and clinical outcomes, represented through a Directed Acyclic Graph (DAG). The SCM applies intervention-based principles to estimate both direct and total causal effects of specific metabolic changes on cognitive and functional measures like MMSE and FAQ scores. This enables a clear, mechanistic understanding of how regional brain dysfunction contributes to AD progression.
- A Causal mapping between brain regions and cognitive tasks is established to uncover how disruptions in localized brain activity contribute to measurable changes in clinical scores. This mapping provides interpretable pathways that link specific metabolic alterations to cognitive and functional impairments. It also facilitates targeted hypothesis generation and informs the design of region-specific diagnostic or therapeutic strategies.
- Counterfactual simulations and placebo analyses were used to validate the robustness of the inferred causal effects, ensuring that the selected regions represent meaningful causal drivers rather than spurious associations.
- A deep learning architecture, the FDG-PET-based Cognition Prediction Network (FDG-PET-CogNet), was designed to predict cognitive and functional scores from brain metabolism data. FDG-PET-CogNet incorporates a feature-wise attention mechanism that dynamically assigns weights based on the relevance of each feature. This allowed the model to focus on clinically meaningful brain regions, enhancing its ability to learn task-specific patterns for MMSE and FAQ score prediction.

This integrated framework combines causal inference with deep learning to provide a structured approach for linking PET image-derived metabolic patterns to clinical outcomes. By identifying brain regions where metabolic dysfunction has a direct impact on cognitive and functional performance, the framework uncovers meaningful biomarkers that go beyond statistical association. These insights enable early and more accurate diagnosis, guide the development of personalized, region-specific interventions, and support targeted monitoring of disease progression, ultimately advancing precision medicine in AD management.

## Methods

### Proposed pipeline for PET image-based cognitive and functional score prediction

**Fig. 1:**
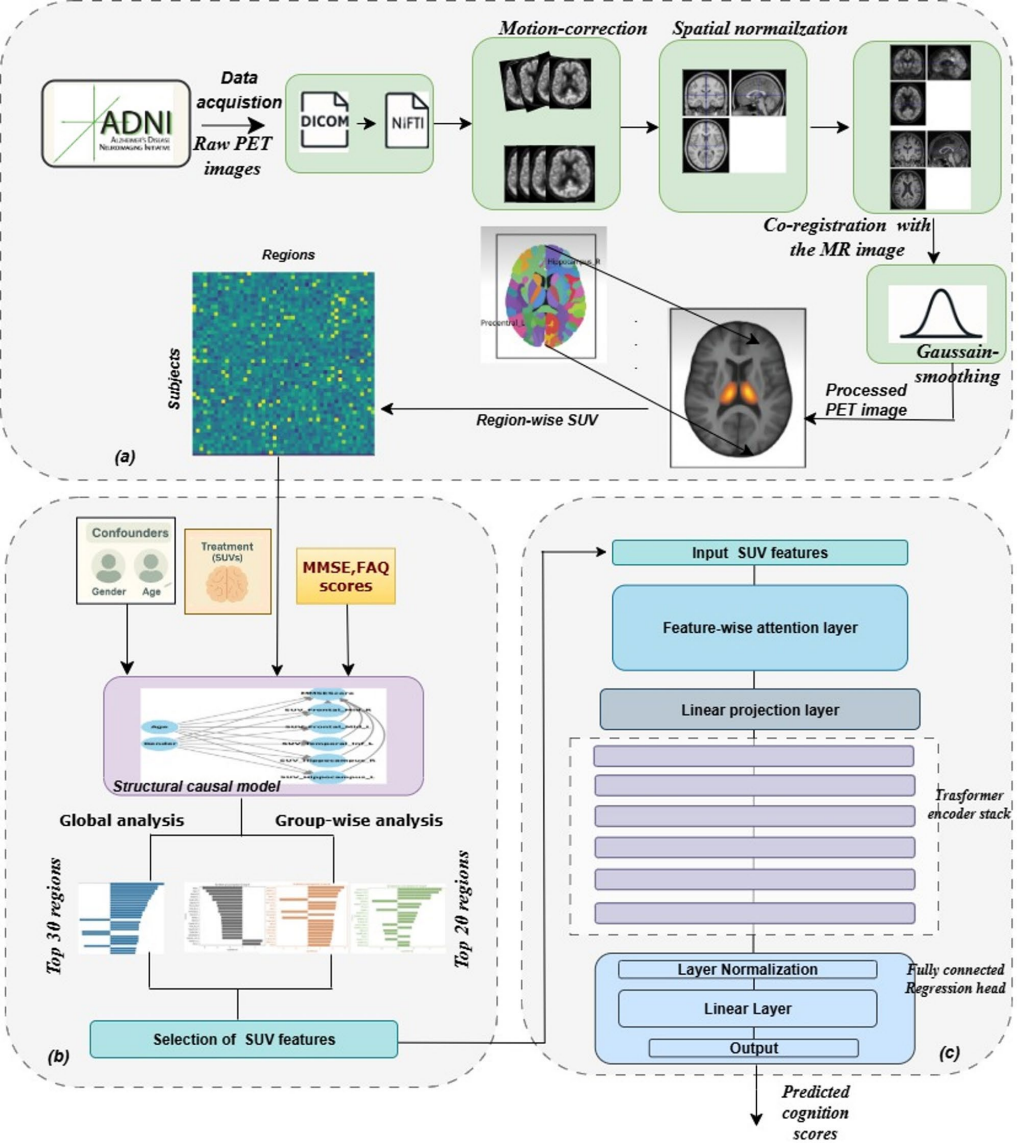
Overview of the proposed framework. (**a**) PET image pre-processing pipeline. (**b**) Causal analysis strategy for the regional impact of SUV on cognition. (**c**) FDG-PET-CogNet model architecture.

The overall workflow of the proposed framework is outlined in Fig. 1. The pipeline begins with comprehensive PET pre-processing to ensure spatial alignment and cross-subject consistency. The steps involved in pre-processing are shown in Fig. 1a, and a brief is given in Sect. "Proposed pipeline for PET image-based cognitive and functional score prediction" (PET image pre-processing). After the preprocessing, Standardized Uptake Values (SUVs) are extracted by computing the mean metabolic activity within each of the 120 brain regions defined by the Automated Anatomical Labeling 2 (AAL2) atlas, resulting in a structured representation of region-wise metabolism. Next, a causal analysis is conducted to identify brain regions whose metabolic activity directly affects cognitive outcomes, specifically MMSE and FAQ scores. A Structural Causal Model (SCM) is implemented using backdoor adjustment to account for confounders like age and gender. This analysis is performed on both the whole cohort and within diagnostic subgroups, including AD, MCI, and Normal Control (NC). The process of causal analysis and region identification is shown in Fig. 1b. Brain regions are then ranked based on their estimated causal effects, and the top-ranked regions are selected as features for predictive modeling (FDG-PET-CogNet). Finally, these causally selected regions are used as an input to FDG-PET-CogNet, a regression model that dynamically assigns importance to each region’s metabolic activity using a feature-wise attention mechanism. This design enhances both predictive accuracy and model interpretability by grounding the learning process in biologically meaningful, causally validated regions. The architecture of FDG-PET-CogNet is illustrated in Fig. 1c.

### PET image pre-processing

All PET images were processed through a standardized FDG-PET pipeline implemented using a custom MATLAB R2023a script with SPM12. The objective of pre-processing was to correct motion, achieve anatomical uniformity across subjects, and ensure reliable SUV quantification. First, the raw DICOM scans, consisting of 6 dynamic frames, were converted to NIfTI format using dcm2niix, preserving all necessary metadata. Motion correction was then applied to align each frame with the first reference frame using a 6-DOF rigid-body transformation. The motion-corrected images were spatially normalized to the MNI152 standard template using a two-step method: first, an affine transformation for global alignment, followed by a non-linear deformation field (ϕ(x)) to accommodate local anatomical differences. Next, each normalized PET image was co-registered with its subject-specific T1-weighted MRI to achieve precise anatomical alignment between functional and structural data. Lastly, a Gaussian smoothing with a 6 mm FWHM kernel was implemented to enhance the signal-to-noise ratio, minimize spatial artifacts, and standardize resolution across subjects for dependable SUV-based regional analysis.

### Data acquisition

**Fig. 2:**
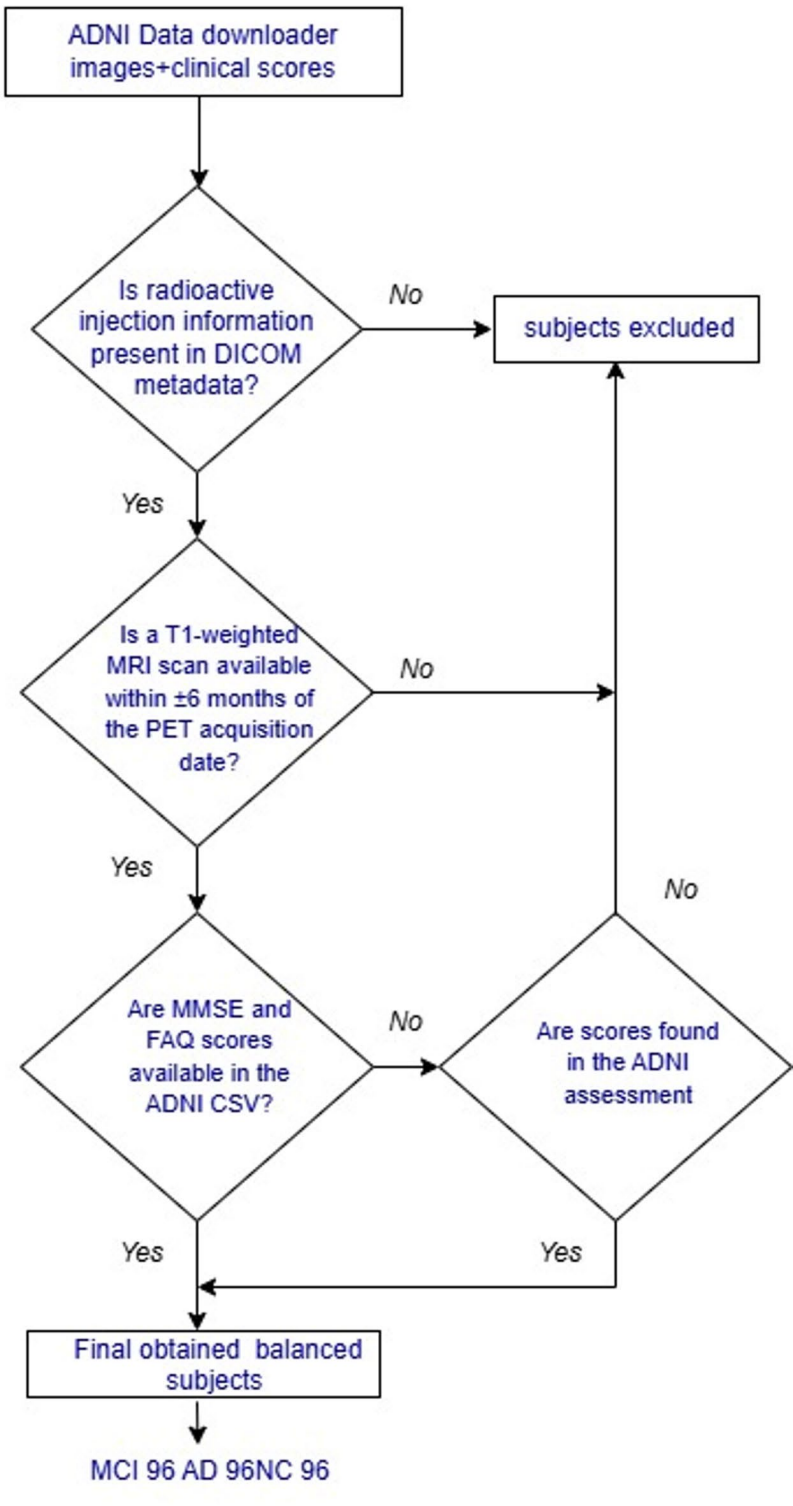
Flowchart of inclusion and exclusion criteria used for subject selection.

For this study, raw 18 F-FDG PET images in DICOM format were obtained from the Alzheimer’s Disease Neuroimaging Initiative (ADNI) database (adni.loni.usc.edu). A structured inclusion–exclusion procedure is shown in Fig. 2.PET images without radioactive injection information were excluded to ensure the reliability of SUV computation. To facilitate accurate co-registration of PET with structural MRI, only MRI scans acquired within a ± 6-month window of the PET acquisition date was selected. Cognitive and functional assessment scores, MMSE and FAQ, are retrieved from the ADNI portal for all subjects.

A total of 288 subjects were selected: 96 with AD, 96 with MCI, and 96 with NC. The diagnostic classifications were based on criteria defined by the ADNI consortium. In the AD group, MMSE scores ranged from 9 to 25, and FAQ scores ranged from 5 to 30. In the MCI group, MMSE ranged from 10 to 28, and FAQ ranged from 0 to 26. MMSE scores ranged from 25 to 30 for the NC group, and FAQ scores ranged from 0 to 5. The complete demographic and clinical characteristics of the cohort are summarized in Table 1.

**Table 1:** Demographic details and clinical characteristics.

Parameter Group	Age Range	Gender (M/F)	MMSE (mean ± SD)	FAQ (mean ± SD)
AD	55.0–90.0	54/41	22.4 ± 3.2	15.0 ± 6.7
MCI	52.0–90.1.0.1	57/39	26.8 ± 3.6	5.4 ± 6.9
NC	59.0–95.0	38/57	28.9 ± 1.4	0.3 ± 1.3

### Extraction of region-wise SUV features

The SUV is a widely adopted quantitative metric in PET imaging that measures the radiotracer uptake in a given tissue, normalized by the injected dose and the subject’s body weight. This normalization facilitates meaningful comparisons of metabolic activity across individuals and brain regions by accounting for physiological variability, like mass and injected activity^20^. Although the Standardized Uptake Value Ratio (SUVR) is more commonly used in clinical PET studies for intersubject normalization, it relies on selecting a reference region assumed to remain metabolically stable. In the context of AD, such an assumption may not always hold, as widespread metabolic alterations can affect even the commonly used reference regions^21^. Therefore, in this study, we adopted SUV as a more direct, assumption-free metric of regional metabolic activity, providing absolute quantification of FDG uptake independent of a reference region. The regional SUV computation is given in Eq. 1.

1 $$\:\:\mathrm{S}\mathrm{U}\mathrm{V}=\frac{\mathrm{T}\mathrm{i}\mathrm{s}\mathrm{s}\mathrm{u}\mathrm{e}\:\mathrm{r}\mathrm{a}\mathrm{d}\mathrm{i}\mathrm{o}\mathrm{a}\mathrm{c}\mathrm{t}\mathrm{i}\mathrm{v}\mathrm{i}\mathrm{t}\mathrm{y}\:\mathrm{c}\mathrm{o}\mathrm{n}\mathrm{c}\mathrm{e}\mathrm{n}\mathrm{t}\mathrm{r}\mathrm{a}\mathrm{t}\mathrm{i}\mathrm{o}\mathrm{n}\:}{\left(\mathrm{I}\mathrm{n}\mathrm{j}\mathrm{e}\mathrm{c}\mathrm{t}\mathrm{e}\mathrm{d}\:\mathrm{d}\mathrm{o}\mathrm{s}\mathrm{e}\:\right(\mathrm{M}\mathrm{B}\mathrm{q})/\mathrm{B}\mathrm{o}\mathrm{d}\mathrm{y}\:\mathrm{w}\mathrm{e}\mathrm{i}\mathrm{g}\mathrm{h}\mathrm{t}\:(\mathrm{k}\mathrm{g}\left)\right)}$$

Feature extraction was performed by aligning each pre-processed PET frame with the AAL2 atlas, which divides the brain into 120 anatomical cortical and subcortical regions. SUV values were extracted for each region in each frame based on atlas alignment. To ensure stable measurements, the regional SUV values were averaged across all frames, yielding a single mean SUV value per brain region. This process transformed voxel-level PET data into a structured, 120-dimensional feature vector, representing stable region-wise brain metabolism for each subject. These features were then utilized for causal inference to investigate regional associations with cognitive and functional outcomes, as well as for predictive modeling of MMSE and FAQ scores in AD.

### Region selection based on direct causal effects

Feature selection in neuroimaging aims to identify the most relevant brain regions from high-dimensional data. In this study, causal analysis was used to improve this process by uncovering true cause-and-effect relationships between regional brain metabolism and clinical outcomes, rather than relying on correlations alone^22,23^. Regional FDG-PET uptake was treated as the cause (treatment variable), while cognitive and functional scores (MMSE and FAQ) were treated as the effects (outcome variables). To ensure accurate and unbiased estimates of these relationships, the first step was to identify and adjust for confounding variables.

### Identification of confounders

To identify confounders, relevant variables were initially selected based on established domain knowledge and subsequently verified using a data-driven causal discovery method. Age and Gender were selected as primary confounders because they consistently influence both FDG PET metabolism and cognitive scores in prior studies^7,24^ Other variables, like APOE4 genotype and education level, were also considered. However, APOE4 data were incomplete, and its main effect relates to long-term genetic risk rather than immediate metabolic-cognitive pathways. Education, while linked to higher baseline cognitive performance, has a limited protective impact against rapid decline and reflects early-life cognitive reserve more than current metabolic function^25^. These variables were excluded to maintain sample size and preserve model stability. The Peter Clark (PC) algorithm was chosen for confounder identification because it is a well-established constraint-based method that detects conditional independence relationships and constructs a Directed Acyclic Graph without assuming a specific functional form. This makes it appropriate for high-dimensional neuroimaging data where relationships are complex but must remain acyclic. Using Fisher Z tests at alpha 0.05, the PC algorithm showed directed edges from both Age and Gender to several regional SUV values and to MMSE and FAQ scores, confirming their role as upstream influences. Univariate regression further supported these results, showing significant associations (*p* < 0.05) between Age/Gender and numerous brain regions. Thus, Age and Gender satisfied the *backdoor criterion*, and their adjustment was essential for estimating the direct causal effects of regional brain metabolism.

### Structural causal model (SCM)

To model how regional brain metabolism influences cognitive and functional outcomes, a Structural Causal Model (SCM) was used. This model includes three key elements: (1) A Directed Acyclic Graph (DAG) that maps the direction of causal relationships among variables. (2) A set of rules that explain how each variable is influenced by its direct causes. (3) An understanding of how all variables are distributed jointly in the data. The DAG serves as a roadmap for determining which variables need to be adjusted to estimate actual causal effects. In this study, the backdoor criterion was used. This approach requires adjusting for variables (confounders) that influence both brain metabolism (FDG-SUV) and cognitive outcomes (MMSE, FAQ) to remove bias from non-causal paths. Since Age and Gender were fully observed and identified as valid confounders, and no mediating variables were modeled, the backdoor strategy was appropriate. This allowed for unbiased estimation of the direct effect of regional brain metabolism on cognitive and functional performance.

### Causal effect Estimation

Causal effects were estimated separately for MMSE and FAQ at two levels: whole cohort and group-wise (NC, MCI, AD). A linear estimator was used because it provides stable, interpretable causal effect sizes and serves as a valid implementation of backdoor adjustment within the SCM. The objective was to identify causality rather than make nonlinear predictions, which made linear estimation suitable.

### Causal region selection and validation

Causal effect estimation was conducted separately for MMSE and FAQ outcomes using both whole-cohort and subgroup analyses (NC, MCI, AD). Each analysis generated a ranked list of brain regions exhibiting significant direct causal influence, and the highest-ranking regions were retained as causally informative features for subsequent model training. To address the possibility of false-positive detections, the causal findings were validated through a placebo (falsification) analysis. In this procedure, SUV values were randomly shuffled within each brain region while preserving the overall dataset structure, and the causal effects were recalculated using the same estimation pipeline. The placebo-derived causal scores were substantially lower across most regions, indicating that the effects observed in the original data were not driven by chance. Counterfactual simulations further verified the coherence of the inferred relationships by demonstrating biologically plausible shifts in MMSE/FAQ predictions in response to hypothetical SUV changes, supporting the validity and directionality of the estimated causal effects.

### FDG-PET-based cognition prediction network (FDG-PET-CogNet)

The FDG-PET-CogNet is a deep learning architecture designed specifically for continuous-valued regression on FDG-PET–derived tabular data. It integrates three key components: a Feature-Wise Attention Layer, a Transformer Encoder stack, and a Fully Connected Regression Head. This design is intended not merely for prediction, but to explicitly capture both the individual importance of each metabolic feature and the non-linear interdependencies among them, providing interpretability alongside predictive power.

A major architectural innovation in FDG-PET-CogNet is the Feature-Wise Attention Layer, which dynamically reweights each FDG-PET feature to reflect its contribution to cognitive outcomes. These attention weights are computed using two linear transformations with ReLu activation and Softmax normalisation, as shown in Eq. 2.

2 $$\:\mathrm{A}=\mathrm{S}\mathrm{o}\mathrm{f}\mathrm{t}\mathrm{m}\mathrm{a}\mathrm{x}\left({W}_{2}.\mathrm{R}\mathrm{e}\mathrm{L}\mathrm{u}\left({W}_{1}.X+{b}_{1}\right)+{b}_{2}\right)$$

Where:

X = Input feature vector of shape (b, f), where b is the batch size, and f is the number of features.

$\:{W}_{1},{W}_{2}$​ = Learnable weight matrices that project features to intermediate dimensions.

$\:{b}_{1},$​$\:{b}_{2}$ = Bias vectors added after linear transformations.

ReLu (⋅) = Rectified Linear Unit activation function,

The final attended feature representation is obtained by element-wise multiplication of the original features with the computed attention weights, as given in Eq. 3.

3 $$\:{X}^{{\prime\:}}=X\odot\:A$$

This attention mechanism provides explicit feature-level interpretability, enabling the model to highlight metabolically relevant brain regions, an important advantage over traditional tabular networks and a key justification for the model’s design.

The attended features are projected into a 512-dimensional latent representation through a linear embedding layer and processed by a stack of multi-head Transformer Encoder layers. Each encoder layer includes multi-head self-attention, feedforward sub-layers, and normalization operations. Although transformers are conventionally used for sequential data, in this context, they are adapted to model complex intra-feature relationships within each subject, enabling the architecture to learn subtle metabolic interaction patterns that simpler MLPs or tree-based models cannot capture. Following the transformer encoder, the model employs a fully connected regression head composed of linear layers with ReLu activations and dropout regularization. This component maps the encoded representation to a continuous-valued output, like MMSE or FAQ scores.

By combining a Feature-Wise Attention mechanism with transformer-based interaction modeling and a regression-specific prediction head, FDG-PET-CogNet offers a strong balance between predictive performance, architectural efficiency, and neuroscientific interpretability. Contrary to being simplistic, the architecture is intentionally designed to avoid overfitting on moderate-sized neuroimaging datasets while still capturing meaningful metabolic dependencies. This task-oriented design differentiates FDG-PET-CogNet from existing tabular models and justifies its use and development in this study. Figure 1c illustrates the complete architecture details.

#### Model configuration

Causal analysis was conducted using GoogleColab. Deep learning experiments were implemented in PyTorch and executed on a GPU-enabled environment. The dataset was split into training and test sets at a 90:10 ratio. Training was carried out using the AdamW optimizer with a learning rate of 5 × 10^−^⁵ and a weight decay of 1 × 10^−^⁸. The Huber Loss (SmoothL1) was used as the loss function, selected for its robustness to outliers. Gradient clipping was applied to stabilize training with a maximum norm of 1.0. A dropout rate of 0.3 was used between layers for regularization.

## Results

### Causal analysis between FDG-PET-derived metabolic activity and MMSE scores

Causal scores derived from PET images are used to infer the direction and strength of causal relationships between brain regions and the MMSE score. Positive scores indicate regions that enhance cognitive function, while negative scores imply detrimental associations. To gain a comprehensive understanding of these relationships, both a whole-cohort analysis and a group-wise analysis were conducted.

#### Whole-cohort causal analysis -MMSE score

Based on the whole-cohort causal analysis, features were ranked by their direct effect estimates, and the top 30 most influential regions were selected for further investigation. Among these, the temporal lobe emerged as a central player, with several areas demonstrating high causal importance. The Inferior Temporal Gyrus(ITG) emerged as an influential contributor, demonstrating direct effect scores of 3.68 on the left hemisphere and 3.22 on the right, emphasizing its pivotal role in semantic processing and object recognition. The left ITG is functionally associated with tasks such as object naming, comprehension of verbal commands, and reading comprehension, all of which are directly evaluated in MMSE subtests. In contrast, the right ITG complements these functions by supporting visual semantic integration and nonverbal memory processes^26,27^. Similarly, the Middle Temporal Gyrus (MTG), a key component of the ventral language stream, showed substantial causal effects (left: 2.99, right: 2.83). The left MTG contributes to lexical semantic retrieval (the ability to find and recall words and their meanings), which is relevant to MMSE tasks like object naming, Immediate Recall, and Delayed Recall. The right MTG, conversely, is implicated in integrating auditory input with verbal sequence processing and episodic memory, functions that are engaged during MMSE Delayed Recall and Repetition tasks^28^. The Temporal Pole also ranked high in causal importance, with 2.52 (left), 2.75 (right), 2.06 (superior left), and 1.96 (superior right). The left Temporal Pole supports autobiographical and semantic memory for tasks like Orientation, Naming, and Delayed Recall, while the right Temporal Pole aids emotional awareness and contextual understanding, relevant for Reading and following instructions^29^.

The Hippocampus, a central memory structure, showed notable output (2.11 left, 1.91 right), aligning with its role in memory encoding and retrieval, particularly relevant for MMSE recall tasks^30^. In the parietal lobe, the Angular Gyrus (3.25 left, 3.03 right) emerged as a key player in sensory integration, arithmetic, and spatial reasoning. Specifically, its left side aids symbolic reasoning, while the right enhances visuospatial awareness, with both contributing to MMSE Orientation and Calculation tasks. Similarly, the Inferior Parietal Lobule (IPL) showed strong bilateral effects, supporting multisensory integration and attention, crucial for drawing, calculation, and orientation in the MMSE^31^.

Beyond the temporal and parietal structures, other brain regions also demonstrated significant causal influence on cognitive outcomes. The Cingulate Cortex (2.61 left, 2.41 right) supports emotional regulation, memory retrieval, and attention, aiding MMSE tasks like Orientation and Delayed Recall^11^. Further supporting memory and spatial recall, the Parahippocampal Gyrus helps in associative and contextual memory retrieval^32^. The Supramarginal Gyrus (right, 1.96) contributes to phonological processing and attention, relevant for tasks like spelling “WORLD” backward and serial subtraction. The Orbitofrontal Cortex (OFCLat_R: 2.34), known for executive functions, impacts MMSE items that require judgment and planning^33^. The Caudate Nucleus (Caudate_L: 1.73) facilitates motor control and attentional shifts, which are relevant during command execution and writing^34^.

**Fig. 3:**
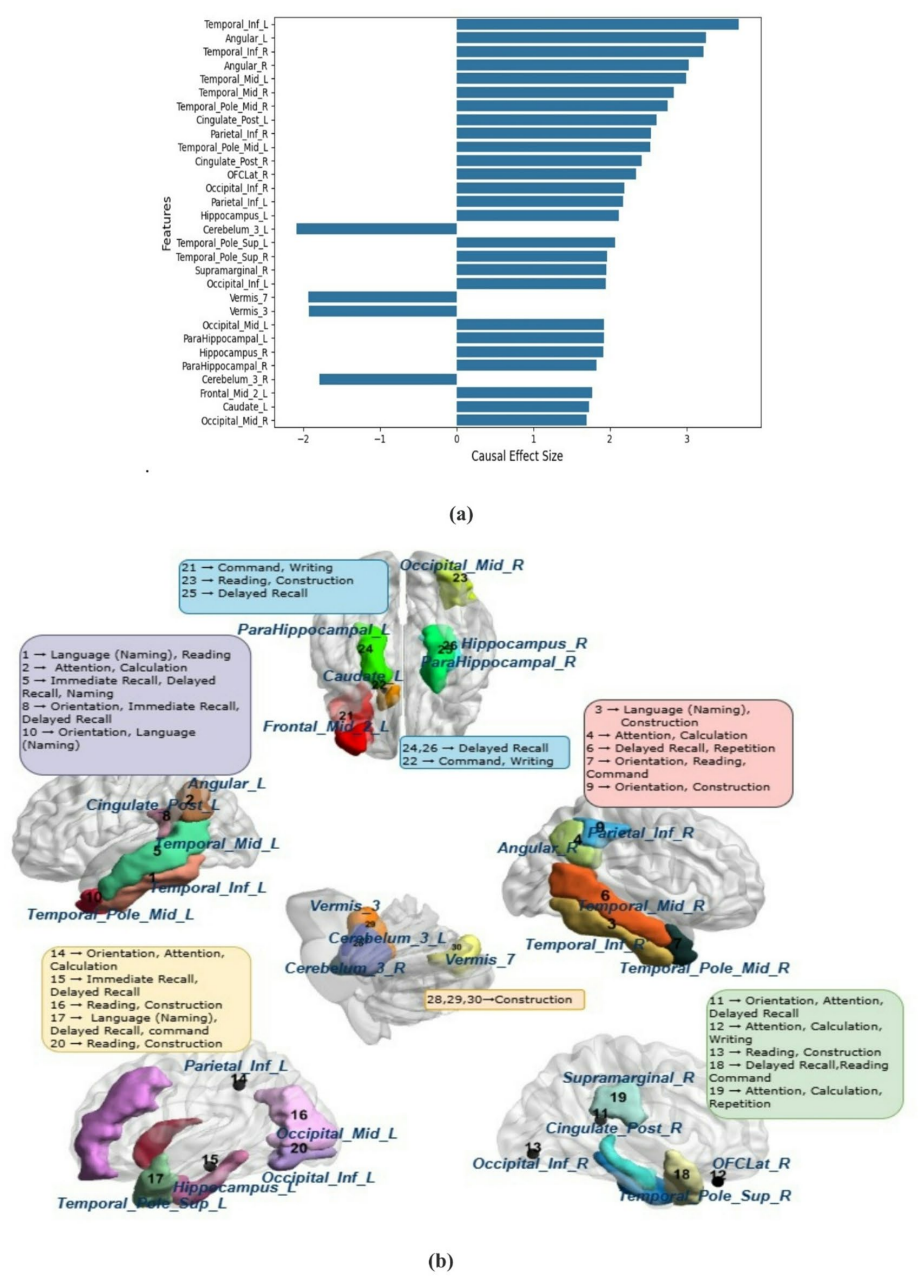
(a) Causal scores of the top 30 features for whole cohort analysis. (b) Causal mapping of the brain region with the MMSE task.

Visual processing regions in the occipital lobe (Inferior: 2.0/1.9; Middle: 1.9/1.7) are essential for reading and copying figures. Frontal areas assist with executive functions, which are critical for tasks involving attention, sequencing, and writing^35^. Interestingly, the cerebellum, including Lobule III (−2.09 on the left and − 1.79 on the right) and the Vermis (−1.92 and − 1.93), exhibited negative scores, indicating impaired motor coordination that may hinder performance on MMSE tasks requiring precise movements like pentagon copying on the construction task^36^.The distribution of causal scores across the top 30 regions is presented in Fig. 3a, while Fig. 3b illustrates the mapped associations between these regions and specific MMSE tasks.

#### Group-wise causal analysis -MMSE score

**Fig. 4:**
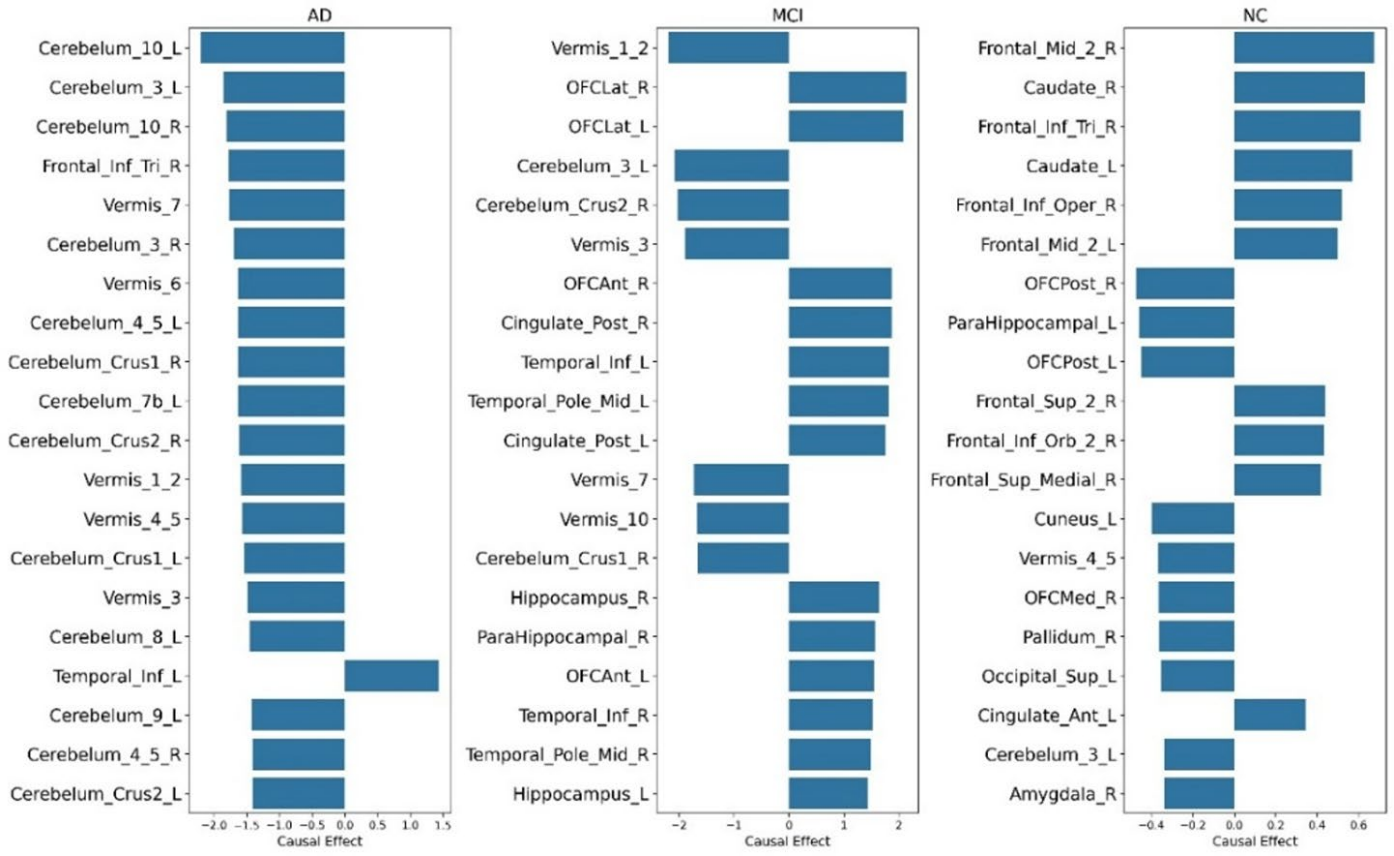
Causal score of the FDG-PET regions for the MMSE score (Groupwise).

The group-wise analysis revealed unique patterns of brain regions engagement across the AD continuum. AD group showed a disrupted causal influence in cerebellar, vermal, and frontal regions that are typically associated with motor control, executive function, and coordination. These disruptions indicate the widespread functional breakdown characteristic of advanced disease. At this stage, compensatory neural mechanisms are largely exhausted. In contrast, the MCI group exhibited a mixed causal profile, with adverse influences from motor-related areas counterbalanced by increased contributions from orbitofrontal, cingulate, and temporal cortices, indicating active neurofunctional reorganization to support memory, attention, and decision-making. These patterns reveal MCI as a transitional phase where targeted interventions may remain effective. The NC group demonstrated balanced and efficient neural functioning. Frontal and basal ganglia regions showed stable contributions to cognition, supporting processes like planning and attention without reliance on compensatory activation. Mild negative influences in posterior or limbic areas appeared within the range of typical variability observed in healthy aging. The top 20 brain regions ranked by their causal scores across each diagnostic group are presented in Fig. 4, highlighting group-specific differences in regional influence.

#### Placebo and counterfactual analysis

Refining the analysis, 35 functional brain zones with high causal strength across the whole cohort and subgroups (AD, MCI, and NC) were identified as critical predictors of MMSE score prediction in FDG-PET-CogNet.The left inferior temporal gyrus emerged as consistently influential across all groups, emphasizing its dominant role. Other regions shared across the whole cohort and multiple subgroups include left and right angular gyri, right inferior temporal gyrus, left and right middle temporal gyri, left and right middle temporal poles, right inferior occipital gyrus, left and right inferior parietal lobules, left and right posterior cingulate gyri, left and right hippocampi, left and right parahippocampal gyri, left middle frontal gyrus (dorsolateral part), and the left and right caudate nuclei. Regions particularly relevant within the MCI subgroup include the left and right lateral orbitofrontal cortices and the left and right anterior orbitofrontal cortices, reflecting subgroup-specific causal influences. In the NC subgroup, the right inferior frontal gyrus, left anterior cingulate gyrus, left and right posterior orbitofrontal cortices, and the right amygdala were prominent, indicating features that help distinguish normal ageing from pathological decline. Finally, several cerebellar and vermian regions including the left and right cerebellar lobule III, left and right cerebellar lobule X, vermis III, vermis VI, and vermis VII were identified as important contributors,, showing predominantly negative causal effects, particularly in AD and MCI groups.

**Fig. 5:**
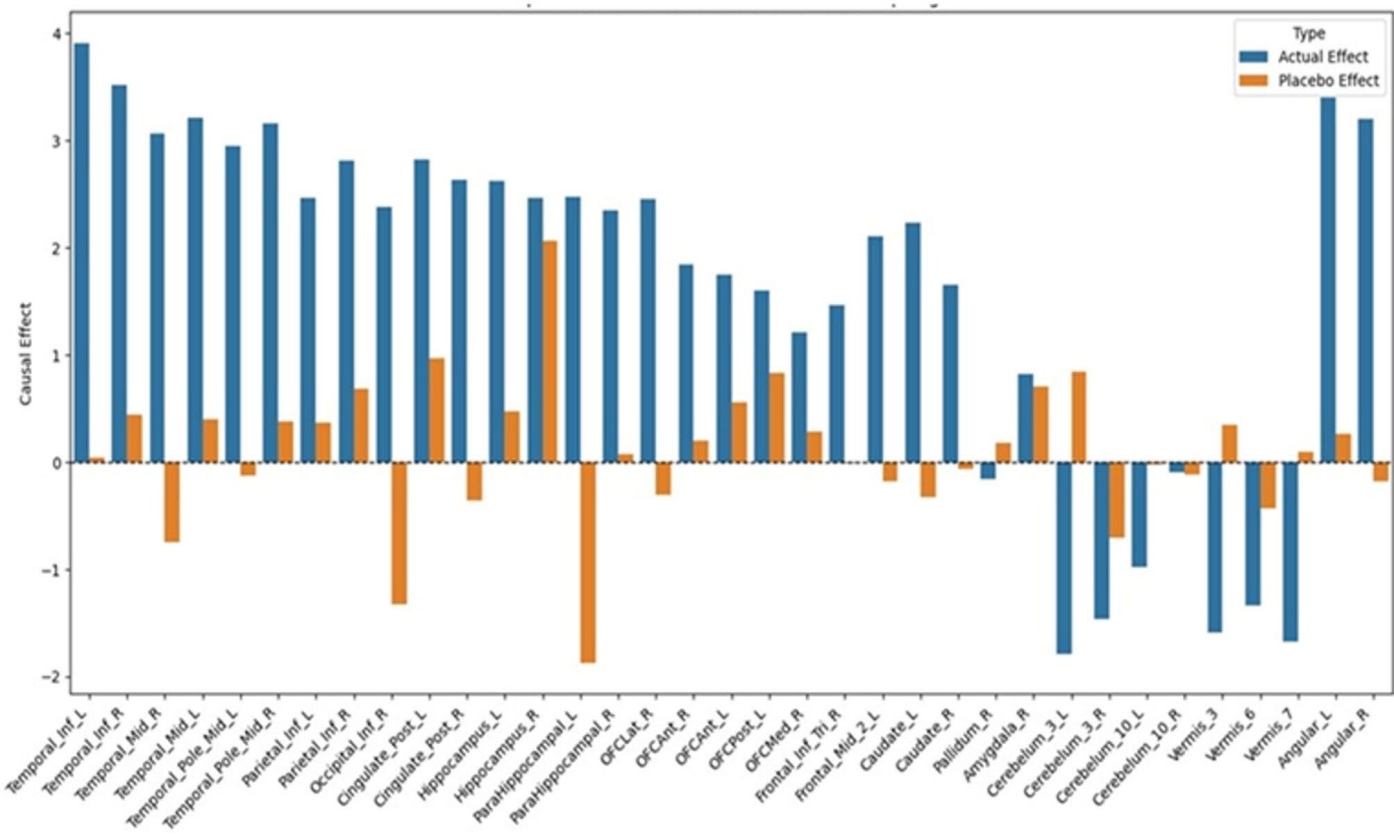
Region-wise comparison of actual and placebo causal estimates influencing the MMSE scores.

To validate the causal inferences, a placebo analysis was performed and causal effects were then recalculated using the same method. The resulting placebo scores were substantially lower across most regions, confirming that the original effects were not due to chance. Even though the biological links are broken by randomly shuffling the data, some regions can still show higher placebo scores due to natural variability and noise in the PET measurements. However, the actual causal effects were significantly greater than those observed under placebo conditions. This is visually demonstrated in Fig. 5, which compares the actual and placebo-derived causal estimates.

**Fig. 6:**
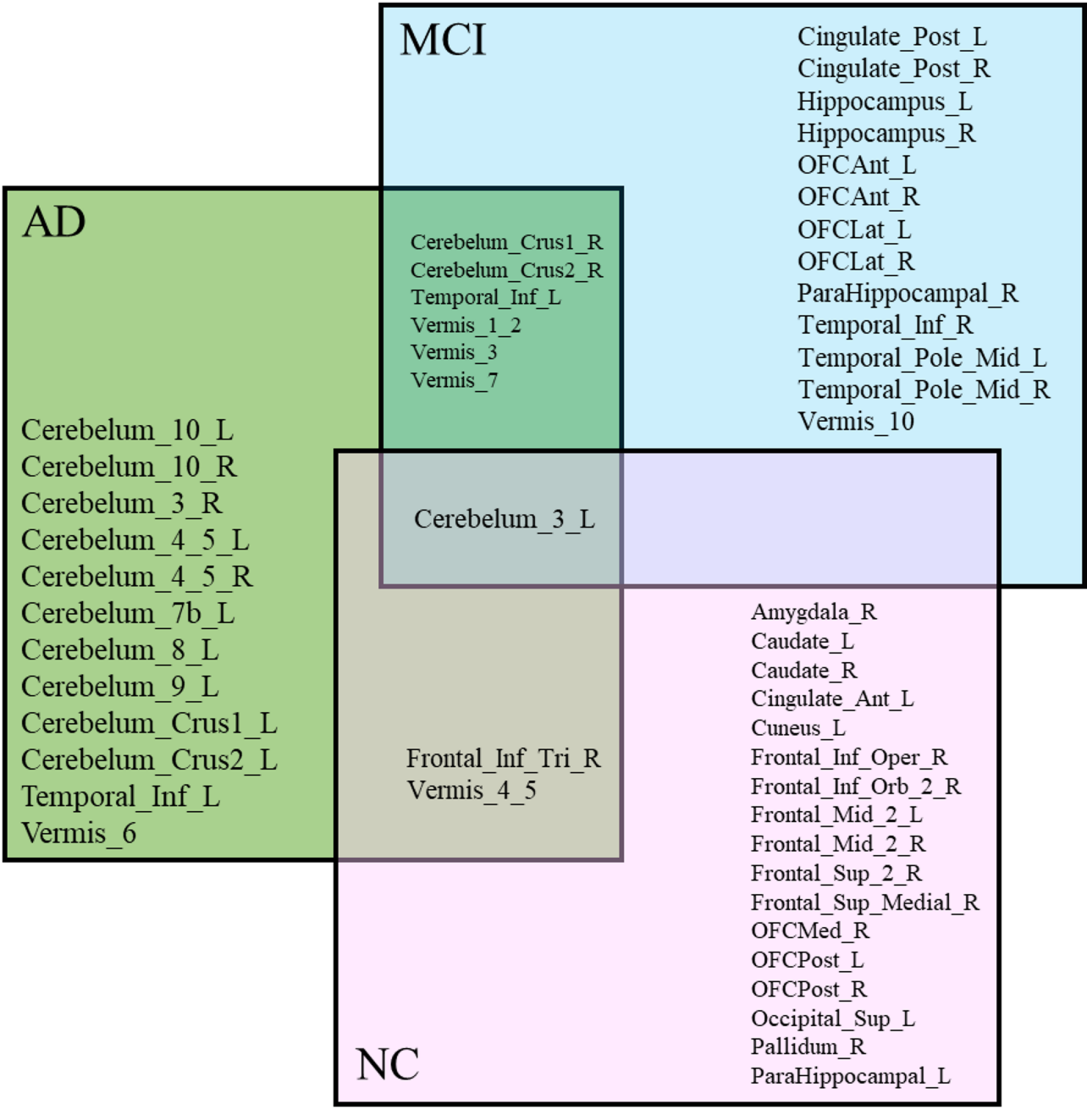
Cross-group overlap of the MMSE-linked brain regions in AD, MCI, and NC groups.

To further understand regional influence, counterfactual simulations were conducted to assess how changes in SUV levels affect predicted MMSE scores. The overlap and divergence among the top 20 brain regions with the highest causal influence on MMSE performance across AD, MCI, and NC groups are illustrated in Fig. 6. Seven regions were common to both AD and MCI, while a few were shared between AD and NC. Each group also showed unique regional patterns.

Notably, Cerebellum_3_L was observed in all three groups and showed a consistent adverse effect, and was selected for detailed analysis. The cerebellum, while traditionally known for its role in motor control, is also involved in a range of cognitive functions, including attention, executive function, memory, language, and visuospatial processing^37^.

The counterfactual analysis of Cerebellum_3_L revealed that increased cerebellar activity is associated with cognitive decline across the AD, MCI, and NC groups. In the NC, increasing cerebellar activity resulted in minimal changes to MMSE scores, suggesting that in a stable brain, the cerebellum’s role remains balanced. However, in MCI and AD, higher cerebellar activity was associated with significantly lower predicted MMSE scores. This trend supports findings from previous studies that cerebellar hyperactivity may initially reflect a compensatory mechanism^38^ but, as the disease progresses, this compensation becomes maladaptive or fails entirely^39^ in AD and MCI.

**Fig. 7:**
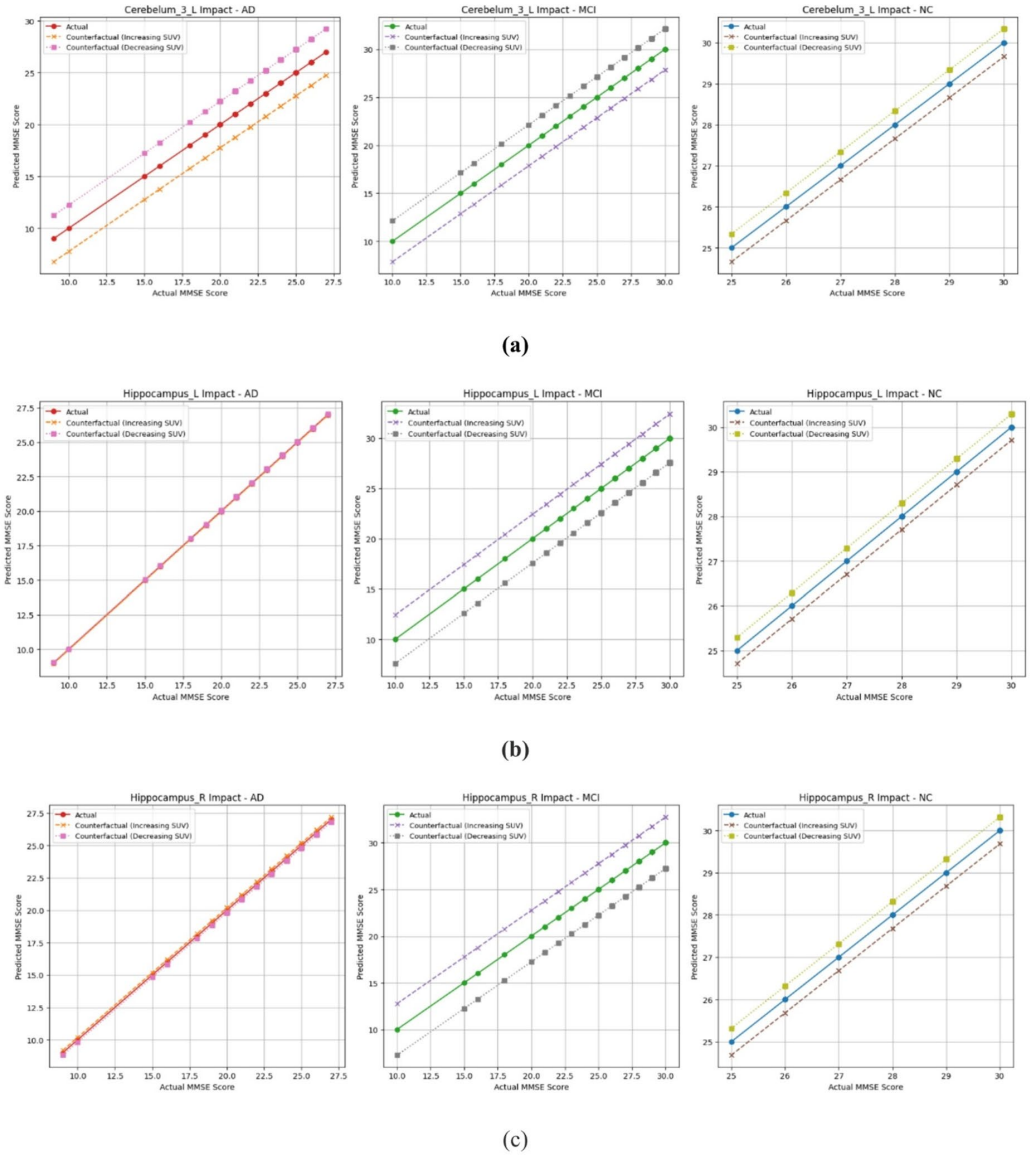
Counterfactual effects observed in AD, MCI, and NC groups. (**a**) Cerebellum_3_L. (**b**) Hippocampal left. (**c**) Hippocampal right.

The Hippocampus, a well-established biomarker for AD^40^ was also examined. In AD, increased activity had little impact, indicating loss of compensatory capacity. MCI subjects showed improved scores with higher hippocampal SUV, suggesting retained function. Interestingly, in NC, higher hippocampal metabolism was associated with a slight drop in predicted cognitive scores, suggesting that excessive activation may disrupt normal memory processing. This is consistent with earlier evidence showing that unusually high hippocampal activity is often seen in groups at increased risk for AD, such as individuals with amnestic MCI. That overactivation in specific hippocampal circuits can actually contribute to cognitive problems (Bakker et al., 2012). These findings indicate that hippocampal hyperactivity may reflect a dysfunctional state rather than improved performance^41^. Figure 7 illustrates the influence of changes in SUV for the cerebellum_3_L and hippocampus regions on the predicted MMSE scores for the AD, MCI, and NC groups using a counterfactual analysis.

### Causal analysis between FDG-PET-derived metabolic activity and FAQ scores

Causal inference applied to FDG-PET data provided critical insights into how regional brain metabolism influences functional independence, as measured by the FAQ. Since lower FAQ scores represent better functional status, brain regions with negative causal scores were deemed particularly important, indicating that higher metabolic activity in these areas supports everyday functioning.

#### Whole-Cohort causal analysis -FAQ scores

Among the most prominent regions, the angular gyrus bilaterally (Angular_L: −8.26, Angular_R: −7.63) and the supramarginal gyrus (Supramarginal_L: −4.95, Supramarginal_R: −5.41) displayed strong negative causal scores, highlighting their essential role in numerical reasoning, memory, and decision-making. These areas are actively engaged in daily FAQ activities like writing checks, settling bills, and keeping track of checkbook balances, all of which necessitate numerical skill and cognitive adaptability^42^.

Extending beyond parietal structures, the temporal pole region, the inferior and middle temporal gyri (Temporal_Inf, Temporal_Mid) also played a substantial role. These regions support semantic memory and language comprehension, enabling individuals to perform tasks such as remembering appointments, managing medications, and understanding financial concepts independently. Adjacent to these, the temporal pole regions (Temporal_Pole_Mid, Temporal_Pole_Sup) were implicated in integrating semantic content with autobiographical memory and social-context processing, which are essential for recognizing routines, planning multi-step activities, and interpreting daily scenarios. The posterior cingulate cortex (Cingulate_Post) supports self-referential thinking, episodic memory retrieval, and attention regulation, functions that are essential for FAQ abilities such as remembering appointments, maintaining daily routines, navigating familiar routes, and managing medication schedules^44^. The inferior parietal lobules (Parietal_Inf_L: −5.79, Parietal_Inf_R: −6.40) are involved in visuospatial reasoning and numerical estimation, contributing to tasks like reading bank statements, interpreting graphs, or calculating expenses^45^.

Memory-related areas like the hippocampus (Hippocampus) are crucial for recalling appointments, recent events, and daily tasks, with dysfunction leading to forgotten due dates or missed routines^30^. The parahippocampal cortex (ParaHippocampal) supports contextual memory and spatial navigation, required for organizing and remembering information while navigating routes or completing tasks like turning off appliances^12^. Frontal lobe regions, particularly the middle and inferior frontal gyri (Frontal_Mid_2, Frontal_Inf_Tri), govern executive functions like working memory, risk assessment, and decision-making, influencing organizational skills and strategic planning^46^. The superior frontal gyrus (Frontal_Sup_2_L) also contributes to executive functioning, especially in prioritizing complex tasks and maintaining goal-directed behavior across multiple steps, which is essential when managing multi-step activities like organizing documents or planning and preparing meals.

**Fig. 8:**
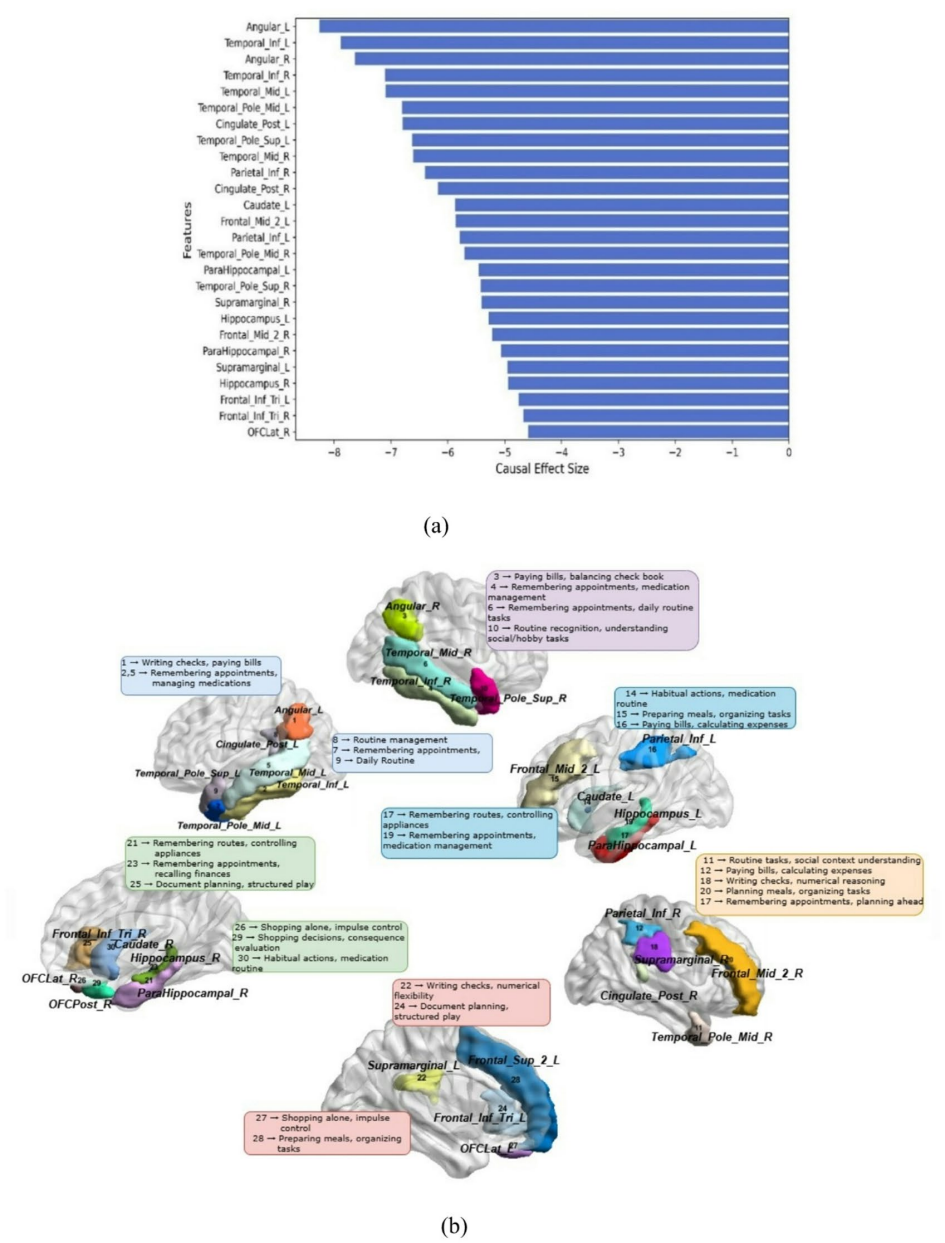
(**a**) Causal scores of Top 30 features for whole cohort analysis.(**b**) Causal mapping of brain region with FAQ task.

The orbitofrontal cortex (OFCLat, OFCPost) regulates emotional decision-making and reward evaluation. Dysfunction in these areas can impair the ability to shop for necessities or handle financial transactions like paying bills, where resisting impulsive choices and evaluating long-term outcomes are necessary. Lastly, the caudate nucleus (Caudate) contributes to motor planning and habit learning, promotes routine, sequential behaviors required for tasks such as managing medication schedules and performing familiar, habitual actions^47^.Details of the causal scores for the top 30 brain regions are provided, and their functional connections with FAQ tasks, based on FDG-PET analysis, are presented in Fig. 8a and b, respectively.

#### Group-wise causal analysis -FAQ score

Following the whole-cohort analysis, groupwise causal analysis identified distinct brain-behaviour relationships across AD, MCI, and NC populations. In the AD group, strong negative causal effects were observed in the Angular Gyrus (L/R) and Parietal Inf Lobule (L/R), indicating significant metabolic dysfunction linked to reduced cognitive and functional capacity. Temporal regions (Temporal Mid L/R, Temporal Inf L/R) and cognitive-motor coordination areas (Vermis_10, Vermis_1_2) also showed strong negative effects. Moderate positive causal values appeared in Cerebellum_10_L, Cerebellum_3_R, and Cingulate_Post_L, possibly indicating compensatory efforts to preserve functionality.

In the MCI group, notable cerebellar involvement was observed, with Cerebellum_Crus2_R and Cerebellum_3_L showing the highest positive causal scores, suggesting early-stage adaptive processing. Additional cerebellar regions (Vermis_1_2, Vermis_3, Cerebellum_Crus1_R) also positively contributed to maintaining independence^48^. However, negative causal effects emerged in Temporal_Pole_Sup_L/R, Postcentral_R, and Occipital_Mid_R, pointing to early functional impairments in memory, sensory integration, and visual-spatial processing.

**Fig. 9:**
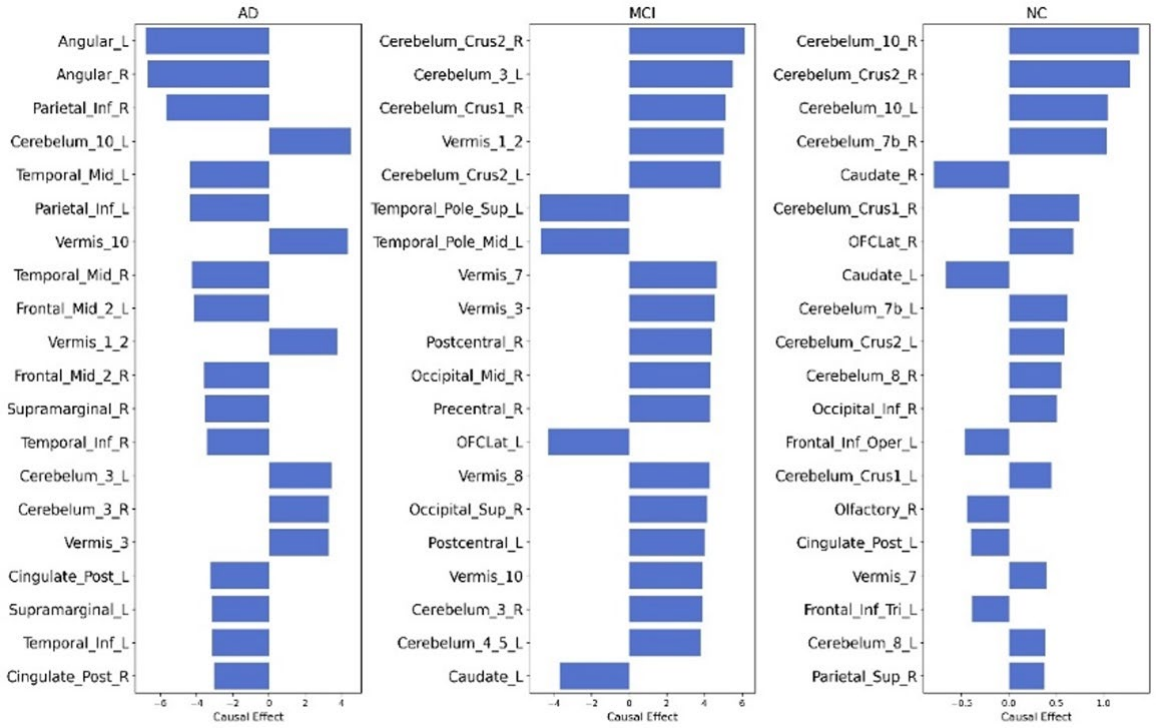
Causal score of the FDG-PET region for the FAQ score (Groupwise).

In the NC group, a supportive profile emerged, with strong positive causal effects in Cerebellum_10_R, Cerebellum_Crus2_R, and Cerebellum_10_L, emphasizing procedural learning and sensorimotor control^49^ The caudate nucleus supported motor responses and cognitive flexibility, essential for multitasking and goal-directed behavior^50^. Robust involvement of prefrontal and orbitofrontal cortices (OFC_Lat_R, Frontal_Inf_Oper_L, Frontal_Inf_Tri_L) supported executive functioning and emotional stability^51^. Robust involvement of prefrontal and orbitofrontal cortices (OFC_Lat_R, Frontal_Inf_Oper_L, Frontal_Inf_Tri_L) supported executive functioning and emotional stability. Occipital_Inf_R, Parietal_Sup_R, and Cingulate_Post_L contributed to visual-spatial cognition and internal awareness. Vermis_7 and Cerebellum_8_L highlighted the integration of emotion and motor functions in daily activities. Figure 9 shows the causal score of the top 20 features group-wise for FAQ scores.

#### Placebo and counterfactual analysis

The identification of 35 key brain regions, from an initial set of 120, was driven by their consistently high causal strength observed throughout the multi-level analysis, including whole-cohort, AD, MCI, and NC subgroup examinations. These regions include left and right caudate nuclei, the left and right posterior cingulate gyri, the left and right angular gyri, the left inferior frontal gyrus (triangular part), the left and right middle frontal gyri, the left and right lateral orbitofrontal cortices, the right inferior parietal lobule, the left and right supramarginal gyri, the left and right inferior temporal gyri, the left and right middle temporal gyri, the left middle temporal pole, the left superior temporal pole, and the right angular gyrus.

**Fig. 10:**
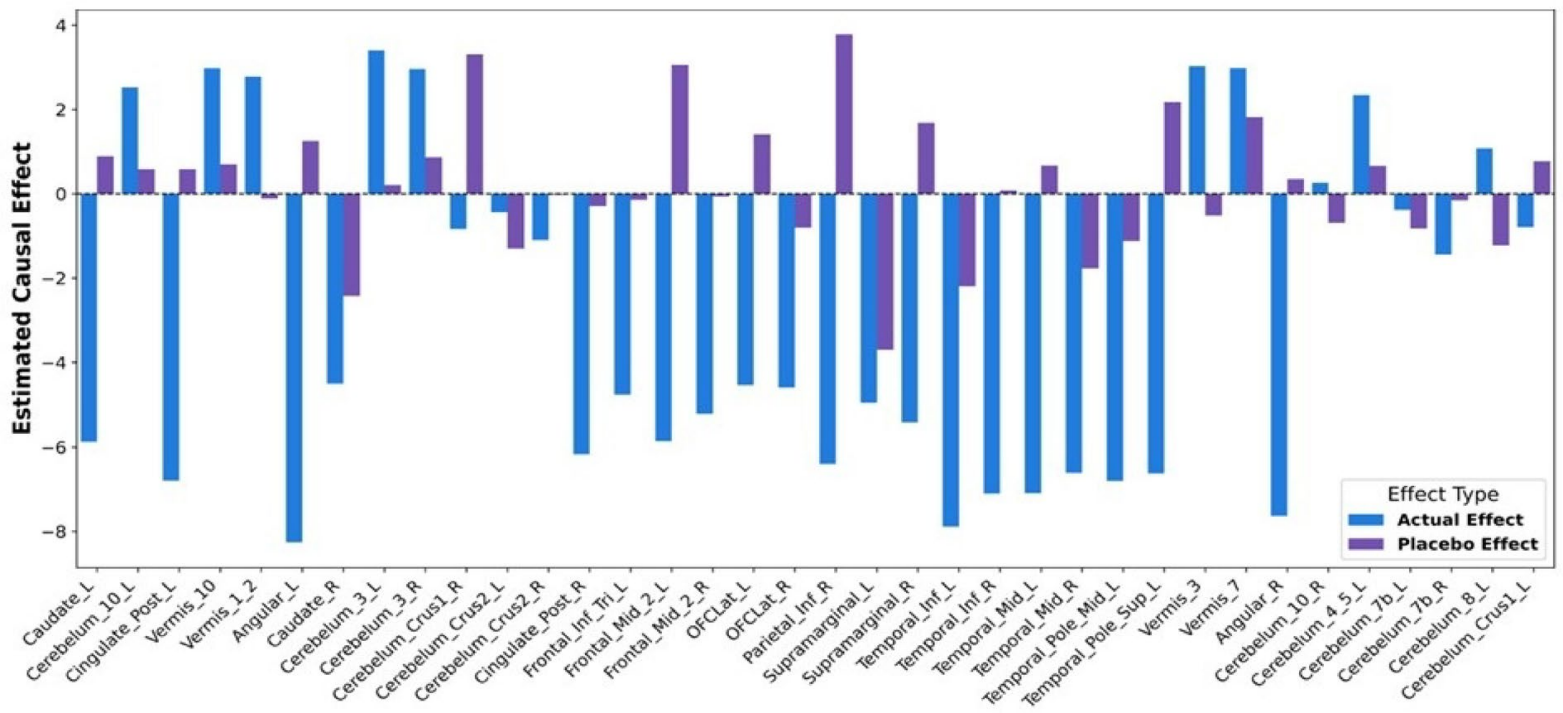
Region-wise comparison of actual and placebo causal estimates influencing the FAQ Scores.

In addition, several cerebellar and vermian regions were identified, including the left and right cerebellar lobule III, left cerebellar lobule IV–V, left cerebellar lobule VIII, left and right cerebellar lobule X, the left and right cerebellar Crus I, the left and right cerebellar Crus II, left and right cerebellar lobule VIIb, as well as vermis I–II, vermis III, vermis VII, and vermis X. Their consistent relevance across the groups underlines their importance in supporting everyday functional abilities, positioning them as a strong feature for inclusion in predictive models. To test the stability of these findings, a placebo test was conducted by randomly shuffling SUV values within each region while preserving the data structure. The causal effects under actual and placebo conditions, as depicted in Fig. 10, showed clear separation across most regions. The actual effects remained robust and predominantly negative, reinforcing the validity of the selected regions. Minor placebo signals, observed in only a few regions, were negligible compared to the real estimates and were probably due to PET scanner noise.

**Fig. 11:**
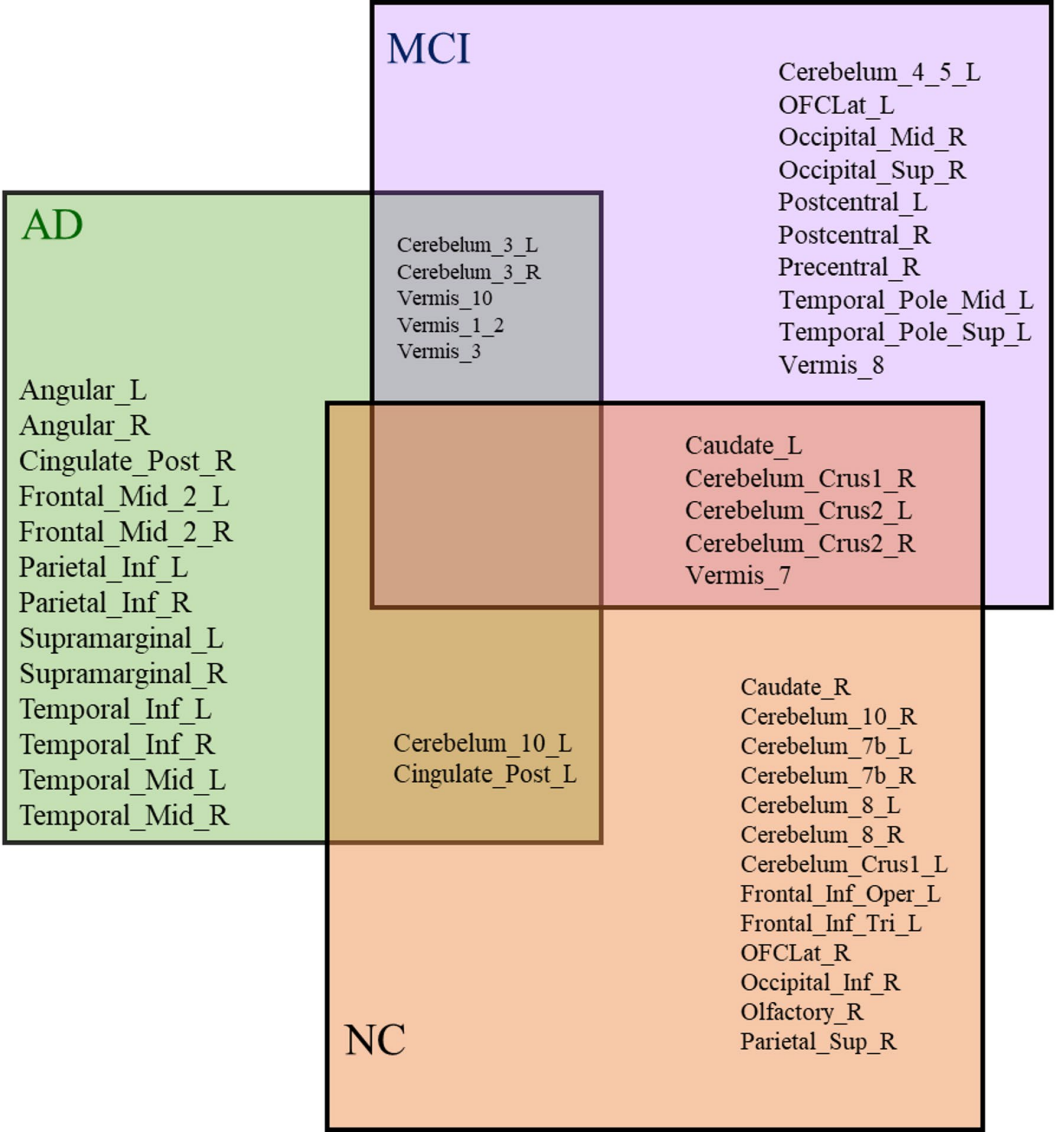
Cross-Group overlap of the FAQ-Linked Brain Regions in AD, MCI, and NC groups.

After validating the key brain regions using placebo analysis, a counterfactual analysis was performed to examine how changes in regional metabolic activity (SUV) influence daily functional ability, as measured by FAQ scores. The overlap and divergence among the top 20 brain regions with the strongest causal influence on FAQ performance across AD, MCI, and NC groups are shown in Fig. 11. No brain region was common to all three groups (NC, MCI, and AD), but several regions were shared between pairs of groups. Cerebellum_3_L, Cerebellum_3_R, Vermis_10, Vermis_1_2, and Vermis_3 were shared between AD and MCI, suggesting their role in early to moderate stages of decline. Cerebelum_10_L and Cingulate_Post_L were common to AD and NC, possibly reflecting early compensatory mechanisms. Meanwhile, Cerebellum_Crus2_R, Cerebellum_Crus2_L, Cerebellum_Crus1_R, Vermis_7, and Caudate_L were shared between MCI and NC, suggesting a potential role in the early stages of cognitive change. These findings indicate that the influence of brain metabolism on functional outcomes varies across disease stages.

**Fig. 12:**
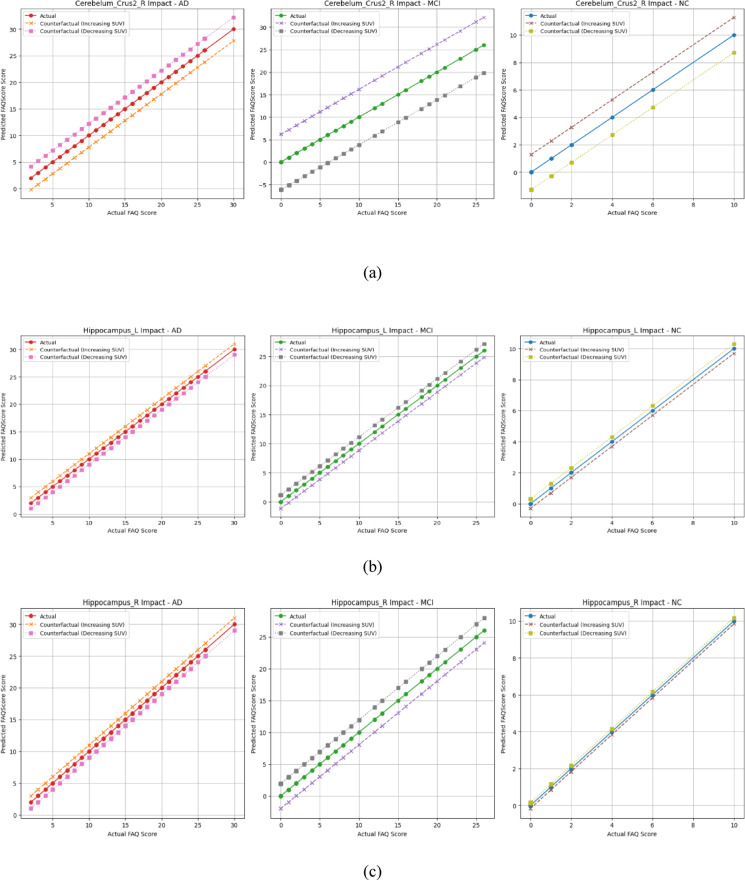
Counterfactual effects observed in AD, MCI, and NC groups. (**a**)Cerebellum_Crus2_R. (**b**) Hippocampal left. (c)Hippocampal right.

The counterfactual analysis revealed distinct influences of Cerebellum_Crus2_R, Hippocampus_L, and Hippocampus_R on FAQ scores across groups, reflecting disease progression. In AD, higher cerebellar SUV predicted better daily function, suggesting residual compensatory activity. Conversely, in MCI and NC, higher cerebellar SUV indicated poorer function, possibly due to maladaptive neural inefficiency. Increased hippocampal SUV correlated with better function in the NC and MCI groups, aligning with their supportive role in memory and learning during early stages. However, in AD, higher hippocampal SUV was associated with worse outcomes, reflecting advanced neurodegeneration or maladaptive hyperactivity. The influence of SUV alterations in Cerebellum_Crus2_R, Hippocampus_L, and Hippocampus_R on FAQ scores differs across AD, MCI, and NC groups, as illustrated in Fig. 12a and b, and Fig. 12c, respectively.

### FDG-PET-CogNet

**Fig. 13:**
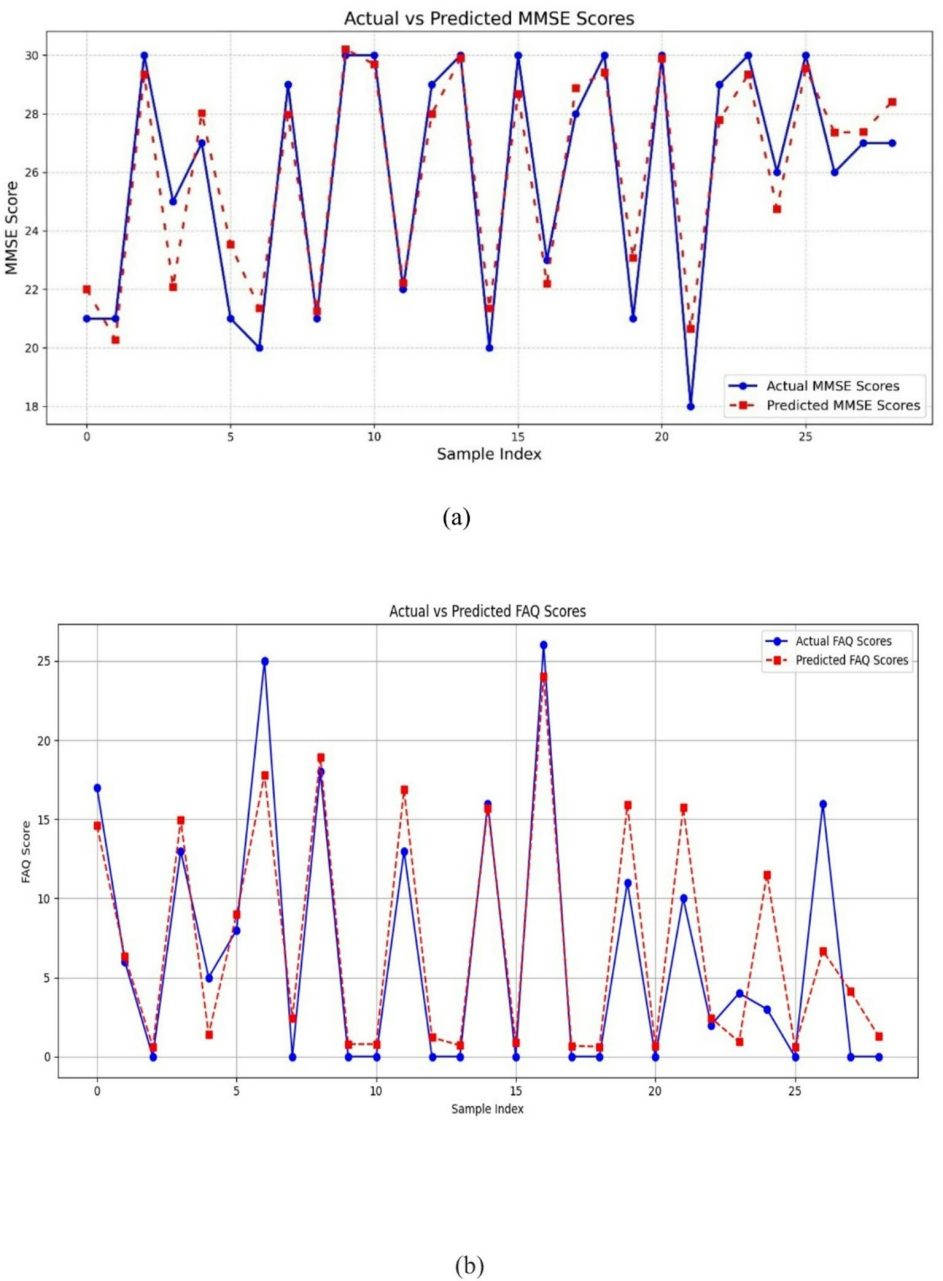
Performance of the FDG-PET-CogNet model—actual versus predicted scores. (**a**) MMSE. (**b**) FAQ.

FDG-PET-CogNet demonstrated strong predictive performance for both MMSE and FAQ outcomes using the brain regions selected through causal analysis. By limiting inputs to causally relevant regions and incorporating demographic factors such as age and gender, the model reduced the risk of overfitting and improved interpretability. The feature-wise attention mechanism further strengthened generalizability by assigning adaptive, biologically meaningful importance weights to the selected regions. For MMSE prediction, the model achieved an R² of 0.90 (MSE = 1.60, MAE = 1.03), showing close agreement between predicted and actual scores. For FAQ prediction, despite the larger inherent variability of functional outcomes, the model maintained high accuracy with an R² of 0.94 (MSE = 3.23, MAE = 0.99). Although external validation beyond ADNI was not performed in this study, the combined use of causal feature selection, structured demographic adjustment, and attention-based weighting provides methodological safeguards against overfitting. The performance of FDG-PET-CogNet in predicting cognitive and functional outcomes is illustrated in Fig. 13(a) for MMSE scores and Fig. 13(b) for FAQ scores.

#### Ablation study

The initial classification experiments were conducted only to identify which model could best handle the FDG PET tabular data. Among all the tested models, the Tab Transformer performed best, indicating that attention-based mechanisms were suitable for this feature space. To reach this conclusion, several machine learning classification models were first evaluated. These models were tasked with categorizing participants into AD, MCI, and NC groups using all 120 imaging characteristics and clinical scores. Traditional models such as Random Forest (58.6%), XGBoost (68%), CatBoost (65.5%), and LightGBM (65.5%) showed moderate performance but were limited in capturing complex nonlinear interactions. Deep learning-based tabular models were then examined: TabNet and SAINT achieved accuracies of 40% and 60%, respectively, while GradTree reached 72%. The Tab Transformer significantly outperformed all other models, achieving an accuracy of 85%. Its strong performance suggests that transformer-based attention mechanisms are effective for modeling dependencies among FDG PET and clinical features.

However, the Tab Transformer is primarily designed for categorical input fields and classification outputs, whereas this study aims to predict numerical cognitive scores. Its embedding strategy, token structure, and classification head are not directly applicable to regression tasks. For this reason, we developed FDG PET CogNet as a regression-oriented adaptation of the transformer approach, incorporating a Feature Wise Attention Layer and a regression head to support scalar cognitive score prediction. Table 2 displays the classification accuracy achieved by each model.

**Table 2:** Classification performance of traditional machine learning and deep learning models.

Model	Accuracy %
Random Forest	58
CatBoost	68
LightGBM	65
Tabnet	40
SAINT	60
Grad tree	72
Tab Transformer	85

## Discussion

This study provides a detailed characterization of how FDG-PET–derived regional brain metabolism is associated with both cognitive performance and functional independence across the Alzheimer’s disease spectrum. By combining whole-cohort and subgroup (AD, MCI, NC) analyses with counterfactual and placebo validation, we demonstrate that neural contributions to cognition and daily functioning are dynamic and disease stage-dependent.

For MMSE prediction, temporal and parietal regions, including the inferior and middle temporal gyri, the angular gyrus, and the inferior parietal lobule, showed consistent effects, especially in the NC and MCI groups. These areas support semantic memory, language, and attention. The hippocampus and parahippocampal regions contributed positively at earlier stages, reflecting their role in memory encoding and retrieval, while their influence decreased or reversed in AD. Cerebellar areas such as Cerebellum III and Cerebellum Crus II also showed early compensatory involvement, whereas greater activity in AD was associated with poorer outcomes, indicating reduced compensatory capacity with disease progression.

Functional independence, as measured by FAQ scores, involved a broader set of regions, including the angular gyrus, temporal poles, posterior cingulate, supramarginal gyrus, and several frontal regions. These areas support executive control, task planning, semantic processing, and the coordination of memory with daily routines. Although there was overlap with MMSE-related regions, areas such as the orbitofrontal cortex, caudate nucleus, and parahippocampal cortex were more specific to FAQ, reflecting their role in motivation and contextual functioning.

The proposed FDG-PET-CogNet model achieved strong predictive performance, with R^2^ values of 0.90 for MMSE and 0.94 for FAQ. The stronger alignment with MMSE reflects the direct dependence of cognitive scores on neural integrity, while the wider variation in FAQ predictions suggests that daily functional abilities are also influenced by non-neural factors.

These preliminary findings may offer a basis for exploring region-guided interventions.For instance, if the left angular gyrus is identified as a key causal region for cognitive decline, targeted neuromodulation such as repetitive transcranial magnetic stimulation (rTMS) could be used to enhance semantic and attentional processing^52^. Similarly, cognitive rehabilitation strategies can focus on tasks known to activate this network. This offers a rational path toward personalized therapy that focuses on the most influential brain circuits.

In addition, the observation that MMSE and FAQ are associated with distinct neural networks suggests the value of outcome-specific imaging biomarkers in clinical trials. For instance, treatments aimed at improving daily function might be evaluated using metabolic activity in frontal, parietal, and cerebellar regions involved in routine task execution. Conversely, cognitive interventions may be better tracked through changes in temporal and hippocampal regions supporting memory and language. This alignment could make it easier to measure whether a treatment is working and ensure that clinical trials focus on the most relevant brain regions for each outcome.

## Limitations and future scope

This study was based on the ADNI cohort, which may limit the generalizability of the findings to broader and more heterogeneous clinical populations. Although age and gender were adjusted for as confounders, other relevant factors, such as education level and APOE4 genotype, could not be included due to incomplete data availability and may be incorporated in future studies. In addition, the present analysis focused on total cognitive scores, whereas future work may also explore individual task-level or domain-specific scores to achieve a more fine-grained and personalised cognitive assessment.

## Conclusion

This study demonstrates the value of FDG-PET-based metabolic profiling in capturing the dynamic, stage-specific neural mechanisms that lead to cognitive decline and loss of functional independence in Alzheimer’s disease. Temporal, parietal, and hippocampal regions primarily support cognition in the early stages, while daily functioning increasingly relies on a broader network involving the frontal, posterior cingulate, and parietal cortices. Cerebellar regions provide compensatory support in MCI but lose this role as the disease progresses, indicating a limit to neural resilience. These findings deepen our understanding of how distinct brain systems contribute to cognition and daily function across the AD continuum. They highlight the importance of early detection and intervention. Integrating targeted therapies based on regional metabolic patterns may provide the most effective window for preserving both cognitive and functional abilities.

## Data Availability

The data used in this study are available in Alzheimer’s Disease Neuroimaging Initiative (ADNI) (adni.loni.usc.edu).
